# Mental Health Literacy of Youth That Have a Family Member With a Mental Illness: Outcomes From a New Program and Scale

**DOI:** 10.3389/fpsyt.2019.00002

**Published:** 2019-02-04

**Authors:** Joanne Riebschleger, Shane Costello, Daniel L. Cavanaugh, Christine Grové

**Affiliations:** ^1^School of Social Work MSU, Michigan State University, East Lansing, MI, United States; ^2^Faculty of Education - Monash, Monash University, Melbourne, VIC, Australia

**Keywords:** mental health, substance abuse, adolescents, children, families, psychoeducation

## Abstract

A program evaluation examined mental health literacy levels and coping outcomes for youth (ages 10–16), before and at the end of their participation in a manualized, school-based mental health literacy program called Youth Education and Support (YES). Most of the youth reportedly had a parent or other family member with a mental health disorder such as depression, anxiety, and/or substance abuse. The mental health literacy levels of program participants from pre to post were evaluated with the developing Knowledge of Mental Illness and Recovery (K-MIR) scale. This scale was validated using item-response theory, demonstrating good psychometric properties. Youth answered two coping questions about their use of positive coping during the program and coping skills compared from pre to post intervention. Findings revealed that youth levels of mental health literacy increased significantly from pre to post program participation. Over 90% of the youth reported an improved use of positive coping strategies from pre to post intervention. The program appeared to deliver enhanced levels of literacy and coping for this sample of youth. The scale appeared to be appropriate to measure youth mental health literacy. Recommendations for practice, policy, and research are offered.

## Introduction

Mental health disorders are one of the most common sources of disability in the world (1). The social, emotional, cultural, and economic costs of mental illnesses affect people across community, business, school, health care, family, and individual sectors (2, 3). Despite the magnitude of the impact, mental illness is an all-too-often a stigmatized topic all over the world. There is a need for mental health literacy programs that provide accurate, non-stigmatized information about mental health disorders and recovery (4). The programs provide practical application of how to seek help for mental health concerns and how to help others who may have mental health symptoms (5).

Children of a parent, or other family member, with a mental illness face particular risks for acquiring a mental health disorder such as depression, anxiety, schizophrenia, bipolar disorder, and/or substance (6). The children, including youth ages 10–16 included in this study, have for too long been “invisible” to services providers of mental health services (7, p. 86). Within individual medical model systems, mental health consumers are rarely if they are parenting minor children. In most of the world, there are little to no mental health literacy programs for youth with a parent or other family member with a mental health disorder (7).

This study is an evaluation of a new youth-focused mental health literacy program. Most of the youth have a parent or other family member with a mental health disorder. The current study includes the psychometrics for a developing scale to measure youth levels of mental health literacy.

## Background

Youth with a parent with a mental illness comprise a large population. One of five people have a mental illness and parenting rates are similar for adults with a mental illness, as compared to adults without a mental illness. England and Sim (8) explored government and private health care databases to find that over 22 million children in the U.S. have a parent with a diagnosis of major depression. Maybery et al. (9) used Australian national health care data to estimate that around 21–23% of young people have a parent with a mental illness.

### Risk and Resiliency

Risk and resilience theorists have provided a theoretical framework to understand how to promote healthy psychological development in individuals that face increased levels of psychosocial adversity (10–15). Youth that have a parent with a mental illness are at a higher risk of developing a mental health illness when compared to their same age peers (16, 17). These youth can be at a higher risk of developing behavioral, developmental, and emotional difficulties (18). Young people of parents with a mental illness may experience school problems and difficulties with attention or self-regulation. For example, they experience higher dropout rates at school (19), an increased likelihood of being taken into foster care (20) and an increased risk of developing a substance abuse disorder (21). Self-harm and suicide rates are higher in young people who have a parent with mental illness (22). Some young people may need to be re-located if their parent is hospitalized and/or very unwell (23). The youth may face increased risk to acquire intergenerational mental illness (24).

Childhood development can be affected adversely when they take on caregiving for parents in a way that is long-term and disproportionate to the child's developmental level of emotional maturity and understanding (25). They can experience negative emotions including shame, fear of conflicts, loneliness feelings of abandonment, sadness, anger, or envy of peers (26). Sometimes, they may harbor resentment toward their parent (27). Often these youth do not receive developmentally appropriate information about mental health (28) and, similarly, do not have the opportunity to develop an adequate and accurate understanding of their parents' mental illness (29).

Not all children are affected adversely by parental mental illness, nor will all children in the same family be affected in the same ways (18). Young people also differ in their beliefs and understanding about the nature of mental illness, its causes and the ways to treat mental health difficulties (30). Darlington et al. (31) suggest that mental illness in a parent does not automatically result in negative outcomes in children. Many parents with mental illness can form healthy attachments and provide nurturing care to their children. Even some situations where children take care of their parents can lead to positive outcomes (25). Caregiving may help the youth build a sense of purpose, potentially supporting resiliency, and reinforcing the parent-child bond (32).

Despite the risks and outcomes that the young people of parents with mental illness may experience, many of these youth demonstrate resilience. Resilience appears when youth engage in positive adjustments to a situation that includes conditions of challenge, risk, and/or adversity (33–35). In general, the more that a young person are seen as resilient, the better their mental health (36). Youth can achieve resilience when there is a balance between adverse events and protective factors. The presence of protective factors can safeguard young people from the impacts of parental mental illness by potentially “buffering” the impact of the risk factors and reduce the vulnerability of a young person acquiring a mental illness. Protective factors include nurturing care by a parent, a close relationship with parents, the child's own problem-solving skills in response to stressors, psycho-education, and accessible social support from peers and other adults (such as teachers) or family members, such as aunts, uncles, grandparents, or siblings (37, 38).

### Stress, Coping, and Adaptation

Another model for considering youth with a parent with a mental illness is stress and coping theory (39, 40). Youth that have a parent with a mental illness may share some of the secondary effects of living in a home where a parent has a mental illness such as underemployment, unemployment, poverty, and/or parental divorce and/or separation (41). Some youth report that it can be stressful living with a parent, sibling, grandparent, aunt/uncle, and other family member with a mental illness as family interactions may change as the family member's illness symptoms can increase or decrease from day to day (41, 42). Further, youth may worry the family may not be able to pay the bills if a parent or other breadwinner is unable to work due to mental illness symptom exacerbation; they may fear others will judge the family member harshly and make fun of the person and, sometimes, the family as a whole (41). They may worry they will inherit a relative's mental illness (43, 44). They may not know how to talk about the relative's mental illness (45).

Coping is part of health and wellbeing, that includes healthy environments, responsive parenting, sense of belonging, healthy activities, resilience, and if mental illness symptoms arise, treatment of the illness (46). Youth can learn to manage stress by engaging in healthy coping activities such as coping self-talk and positive self-talk (47, 48). For example, positive coping behaviors can include exercising, talking to friends, writing, making a craft, and/or listening to music. It can include seeking help for family mental health crises. Stallman (46) notes that youth can make a coping plan to recognize personal and family stress and then engage in stress-reducing coping behaviors. As youth increase their coping behaviors, they can learn to better manage stress and, over time, adapt to stressful situations. They can even move beyond mere adaptation as their crisis plan offers new buffers or protective factors toward youth developmental resiliency (49).

### Mental Health Literacy Interventions and Measures

Mental health literacy interventions, sometimes called psychoeducation, aim to provide accurate, non-stigmatized information about mental illness and recovery to mental health consumers and/or family members. Jorm et al. (50) describe mental health literacy as consisting of several components such as the:

ability to recognize specific disorders, knowledge of how to seek mental health information, knowledge of risk factors and causes, knowledge of self-treatments and of professional help available, and attitudes that promote recognition and appropriate help-seeking (p. 469).

Mental health literacy interventions can teach young people coping skill and strategies to help them reduce stress. They can also respond to specific requests of young people who often ask for more mental health information to help with their understanding about their parents' illness (28, 29). Many young people report that they do not receive enough information about their parent's mental health (28) and are left “guessing” or “figuring out” what is happening to their parent. Some youth may develop misconceptions about mental illness such as blaming themselves for their parent's illness, or believing the mental illness can be caught like a cold or be “passed” onto friends (51). A lack of understanding about parental mental illness hinders the recognition and promotion of appropriate help seeking and reduces the likelihood of young people pursuing help for their difficulties (52). Mental health literacy programs often offer social support; for example, youth may use the mental health information and supportive relationships acquired in mental health literacy programs to manage practical issues such as finding support when their parent is hospitalized and when they need a break from caring for their parent (23, 51).

Clearly, youth would like to be “kept in the loop” and informed about their parents' mental illness (53) and want to talk about their experience (29). However, they may be unsure of the implications of accessing support, such as discussing the impact of stigma for having a parent with mental illness (54). Each young person will process information about his or her parent's illness differently and so will have varying information needs. Young people's understanding of their parent's illness is constantly evolving and changing including the amount and type of information they acquire (55).

Gladstone et al. (29) suggest that psycho-education should attempt to include young people's views of their parent's illness and should recognize the youth's role within the family context. However, it is important to note that the kind of mental health knowledge, and how it is shared with youth, is mostly developed and implemented by adults who decide what youth need (56). Mental health information for youth should be examined from a child-centered approach; there is a need to investigate how mental health information is used by youth and whether this is helpful for the young person, if at all (29). The voices and experiences of young people and their families when developing mental health literacy programs also need to be heard (53, 57).

There are many emerging mental health literacy programs designed to promote resilience in children who are with parents or family members with mental illnesses (7, 18). For example, in Finland (58) and Sweden (59), there are national implementations of a family intervention program to encourage child and parent communication about the parent's mental health condition, recovery strategies, and social service coordination (58). However, in most areas of the world, only a small percentage of youth with a parent or other family members with a mental illness have access to mental health literacy programs. Even in Australia, where there are a number of family-, youth-, and parent- centered programs with mental health literacy content, there is limited access to the programs across many regions of the country. Many of the programs are emerging; they are not yet considered evidence-based (18). A lack of evidence-based programs that provide mental health information and support for youth, parents, and other family members could be a potential barrier to funding. Many private and government organizations prefer to fund evidence-based programs (60).

A lack of mental health literacy scales with sound psychometric properties are a barrier to building evidence-based practices, especially in evaluating interventions for young people with a parent with a mental illness. O'Connor et al. (61) conducted an extensive literature review of mental health literature scales. They found that of the measures they reviewed none covered all of the constructs of mental health literacy, such as disorder recognition, help seeking, risk factor identification, information seeking, causes of mental illness, self-treatment/coping, and risk factors. They also reported that most measures used had methodological gaps in psychometric validation and norming.

Since the publication of that article, new scales with increased rigor have been published (62, 63). The first measure was normed primarily on college students (62). The second measure was normed on a combination of health care practitioners and those in the general population ages 15 and up (63). Additionally, the second measure focused on three specific disorders (schizophrenia, anxiety, and depression). The most rigorous mental health literacy measures available appear to be normed only on adults (62). The work of Kutcher et al. (64) included a scale designed to measure mental health literacy as an indicator of increased mental health resiliency among high school students. However, the reading level appears to exceed that needed for middle school students.

Measures have been used to assess mental health literacy programs delivered to young people with a parent with a mental illness (5, 43, 65) or with young people without a parent with mental illness (66). However, these measures do not appear to capture all of the mental health literacy constructs. They also contain limited information about scale psychometric validation or norming. Therefore, building on these current works in the field comprehensive and robust mental health literacy scales are needed for youth of varying ages (4). Specifically, YES program services providers indicate that their evaluation of the program is need of a measure for youth aged 10–16 that have a parent or other family member with a mental illness. More evidence is needed for youth mental health literacy programs and scales to move the field forward toward building evidence-based practices.

## Methods

This study evaluated the outcomes of the YES mental health literacy program. The guiding research question asked, “What are the pre-post mental health literacy and coping outcomes reported by youth attending the Youth Education and Support program?” Given the lack of psychometrically validated measures of youth mental health literacy (4), the first phase of the evaluation study included development and analysis of an instrument to measure youth mental health literacy. The second part assessed youth participant self-reported levels of pre to post program mental health literacy and coping behaviors.

### The Knowledge of Mental Illness and Recovery (KMIR) Scale

The KMIR scale items includes 36 questions. Eighteen are set in a true and false format. Eighteen use a four-item multiple choice response set. The primary aim of the scale is to assess the mental health literacy of children ages 10–16. The Flesch-Kincaid scale assigned the KMIR a 4.8 reading level. This means the scale is an appropriate reading level for youth 8 months into the fourth grade. In the US, most of these youth would be about 9 years old. Completion time is 11–14 min. The primary investigator used or modified questions found on mental health websites (67, 68), and as found within a nonfiction book written for teens with a parent with a mental illness (69). In addition, the scale author wrote 16 new questions, as the first version of the KMIR scale was pretested initially with six middle school children and who took the test and then offered suggestions for improvement. Their comments led to the second version of the scale administered to 39 YES program youth. Version two was then administered to 216 middle school students in three schools in the Midwest, which was used in the psychometric validation presented below. The validation sample included 55.10% female participants, with a mean age of 13.54 years (standard deviation 0.71 years, ranging from 11 to 16 years). The participants identified as Caucasian (80.10%), Black/African American (6.50%), Latino/Hispanic (6.00%), American Indian (2.30%), Asian American (0.50%), and other or did not answer (2.8%). Following the validation, the scale was administered to the 46 YES participants in the current study.

To determine the validity of the Knowledge of Mental Ill00ness and Recovery (KMIR) were evaluated for goodness of fit to a graded response model using Microsoft R Open version 3.4.2 (70) and R Studio version 1.1.383 (71). The *M*_2_ limited information goodness of fit statistic (72) was calculated for each subtest, with significant results (*p* < 0.01) indicating less than adequate fit. However, when sample sizes are relatively small, a corresponding small Root Mean Square Error of Approximation value (RMSEA < 0.09) suggests that a lack of fit may be due to a limited amount of “model error” (73); common in strong parametric models (72). The Standardized Root Mean Square Residual (SRMR) is the square root of the difference between the residuals of the sample covariance matrix and the 0covariance model (74). Values range from 0 to 1.0, with well-fitting models obtaining values <0.06 (75). Marginal reliability coefficients (analogous to Cronbach's alpha coefficients) are calculated using Lord and Novak's true score model (76).

At the item level, fit was investigated further with *S–X*^2^ item level diagnostics (77, 78); infit and outfit (79, 80); local dependency (81); and differential item functioning by gender (82). Non-significant *S – X*^2^ item values are indicative of good item level fit, while infit and outfit scores between the range of 0.5 and 1.5 are considered productive for measurement. Local dependency indicates a relationship between items where the ability to answer one item is predicated on the ability to answer another item. For example, to demonstrate knowledge of bipolar disorders, one would likely need to be able to demonstrate knowledge of depressive disorders. Local dependency is identified using positive correlation coefficients of 0.20 or greater in a correlation matrix of residuals. Differential item functioning indicates a gender difference in likelihood of correctly endorsing an item for participants of the same underlying ability. One example of differential item functioning would be a question regarding the role of social support in recovery, which is generally answered correctly by females and not by males—reflecting differences in gendered experience and norms rather than knowledge.

The results of the Rasch analyses suggested that aside from a small number of locally dependent items (one item pair in *Mental Illness*; two pair in *Recovery*; and one item pair in *Stigma*), the subscales and total KMIR scales demonstrated reasonable model fit. There were no redundant items, no items demonstrated significant misfit, and no differential item functioning was observed. Infit and outfit statistics were all within the recommended range of 0.5–1.5. The fit statistics are presented in Table 1.

**Table 1:** Rasch model fit statistics for KMIR scale.

	***M_**2**_***	***df***	***p***	**RMSEA**	**SRMR**	**Reliability**
KMIR total score	1199.88	629	<0.001	0.06	0.11	0.87
Mental illness	83.79	65	0.06	0.04	0.08	0.69
Recovery	225.37	65	0.11	0.09	0.13	0.68
Stigma	147.34	65	<0.001	0.06	0.11	0.68

### The Youth Education and Support (YES) Program

Forty-six youth participants were drawn from an urban, Midwest American middle school. Social workers employed by the school visited grade 5–8 classrooms to talk about students' opportunities to participate in the Youth Education and Support (YES) program to learn about mental health and recovery. If considering YES program participation, youth submitted a note of interest later to the mailbox of the social worker. This ensured that interested youth could not be identified as those turning in program inquiries at the time of the social workers visits to the classrooms. School social workers then met with inquiring youth and once their interests were confirmed, communicated with parents to explain the program, and if parents allowed, obtained signed informed consent for participation. The primary investigator trained the school social workers' in obtaining informed assent from youth and consent from parents in accordance with the overseeing university's Institutional Review Board. Parents also completed youth and family demographic information. Demographic information included parents' checking a general category of relative that they identified as having a mental health disorder. At no time were specific individuals identified by name.

Participation in the study did not require that youth have a parent or other family with a mental illness but it was a preferred criteria for inclusion in the program. Prior to the first group session of the YES program, school social workers obtained a pre-intervention youth completion of the Knowledge of Mental Illness and Recovery scale (KMIR) to assess youth beginning knowledge of mental health literacy information. The KMIR scale included knowledge about common mental illnesses, recovery, and stigma.

The Youth Education and Support (YES) program is a manualized, ten-session mental health literacy program for youth in grades 5–8. It was developed by the first author over a 12 year span of time with recommendations from children, youth, and adults that reportedly have a parent or other family member with a mental illness. Recommendations for the content were also drawn from mental health professionals, family members with a mental illness—especially parents, the professional literature, and reviews of existing mental health literacy program curricula from the United States, Australia, England, and Canada. The program continued to be modified over time in response to program evaluation data drawn from the comments of youth participants, their parents, other family members, middle school teachers, fidelity observers, and program facilitators.

Each session is about 50 min so there is around 8 h of total program time. Across the program, the content covers mental health literacy content including knowledge of mental illness in general, including risks/causes; specific mental health disorders, including major depression, types of anxiety, schizophrenia, bipolar disorder, and substance abuse, e.g., alcohol, marijuana, meth amphetamines, inhalants, and opioid addiction. It covers risks for mental illness such as stress and genetics, while emphasizing that it is difficult to know what causes any one individual to develop, or not develop, a mental illness. A holistic recovery model anchors the recovery-focused learning content. Youth discuss how to seek help for mental health concerns and how to help others with these concerns. Mental illness “truths” (facts) and “myths” (stigma) are included regularly within the program.

Words of the day guide session topics that include YES (introduction to the YES program), mental illness, coping, depression, recovery, substance abuse, co-occurring (mental illness and substance abuse), family (impacts of mental illness on family members), planning (for mental health crises and the future of the youth), and graduation (end of group celebration). Each session begins with attendance, word, of the day, and food and drink, i.e., cookies, fruit, cheese, and water. Every session includes a check-in on stress and coping, as well as discussion of ways to manage stress. Some kind of hands-on active learning activity takes place in each session. For example, youth construct a crisis mobile with drawings and names of behaviors that they believe will help them reduce stress and “stay in balance.” They develop a customized individual coping plan with illustrations. They color pictures of a depressed vs. a non-depressed brain based on an MRI example. They engage in a hopscotch-like team competition to plan for ways to work toward their future goals. They watch a movie clip about a family with a parent with a mental illness and discuss what each family member may be feeling and/or thinking. They walk rapidly about the room in a circle demonstrating how substance abuse and depression can have a “circular” effect, sometime leading to increased levels of mental health symptoms.

All of these activities seemed to work well with all of youth, but those ages about 13–16 spent more time on discussion. For youth ages 10–12, facilitators talked at a slightly slower pace and used lower language levels. At each session end, each youth chooses a small item from a “prize box” to reward their participation. Each session ends with a take home educational document to share with parents. For example, some of the parent handouts are how to talk to a child/youth about mental illness and when and how to seek help should a parent have concerns about their son or daughter's mental health. These are general information handouts; no specific youth, parent, or family application is included.

The program was co-led by a school social worker and university faculty member with expertise in mental health practice and research. Sessions took place during the school day at varying days of the week and times of the day to reduce youth participants from missing too much of one class. Almost every session included a trained observer who completed a YES program fidelity scale that measures the extent the session met stated learning objectives. The observer sat in the rear of the classroom and did not participate in programming.

At the last session, facilitators asked youth participants to think about their coping at the beginning of the program. A pause followed for youth reflection. Then they were asked to think about their level of coping at the end of the program. The facilitator emphasized that honest responses were most helpful; youth were encouraged to choose the answer that seemed to fit their experience. Youth reported the extent they thought their pre to post coping met one of the following responses, i.e., a lot worse, a little bit worse, about the same, a little bit better, or a lot better. Facilitators also asked youth to report how frequently they were able to use positive coping behaviors during the time they were participating in the program. Positive coping behaviors are activities that youth choose to deal with their stress that are likely to make the situation better. For example, thinking positive thoughts or playing sports may help some youth reduce their stress. Negative coping behaviors such as hitting a window or yelling at someone are likely to make the situation worse. Youth circled a positive coping frequency estimate of not very often, sometimes or often.

These simple questions were included in the midst of developing the program. This was primarily because the A-COPE, used in early YES program measurement, seemed to rely on youth implementing a wide array of coping measures to score higher in coping (83). Since the YES program focused on implementing one to three youth-selected coping behaviors regularly, combined with relatively short programming time and the fact that the A-COPE scale did not include computer and online coping behavior choices, this simplified coping measurement appeared to capture youth ideas about their coping.

## Results

Forty-six of fifty-two youth, ages 10–16, completed the YES program after development of the 2012, or third version, of the K-MIR scale. Reasons for non-completion were schedule conflict with team sports (*n* = 1), second parent withdrew consent (*n* = 1), not interested in continuing (*n* = 2), moved out of area (*n* = 1), and not known (n = 1). Table 2 notes the demographic composition of the YES participant youth. More than half of the sample were non-Caucasian. Most participants were in grades 6–8. About 59% of the sample reportedly had a relative with a mental illness, most frequently a parent with reported diagnoses of depression, bipolar disorder, or borderline personality disorder. Over 10 percent of the youth reported had a parent with a dual diagnosis of mental illness plus substance abuse (primarily alcohol abuse). Notably, parents reported that over 28% of the youth also had a mental health diagnosis, most commonly ADHD, anxiety, and depression.

**Table 2:** Demographic and household details of the YES program sample (*N* = 46).

**Demographic**	**% or mean**
Female	63.00%
Male	32.60%
Age	13.07 years (SD 0.82)
**RACE**	
Black	21.30%
White	48.90%
Latino/Hispanic	12.80%
Mixed Race/Other	17.10 %
**GRADE**	
6th	19.60%
7th	8.70%
8th	69.60%
Missing	2.20%
**REPORTED FAMILY MEMBER WITH DIAGNOSIS**	
Parent	34.80%
Sibling	15.20%
Grand parent	4.30%
Aunt/Uncle	2.20%
Other family member	30.40%
None reported	30.40%
Missing	10.90%
Child reported to have a diagnosis	28.26%

Table 3 details the distributions of correct answers by item, subscale, and total scale; and statistical comparisons from pre to post intervention.

**Table 3:** Repeated-measure *t*-test of KMIR by item, subscale, and total scale scores.

**Items and scales**	**No. correct pre-program**		**No. correct post-program**		***t***	***p***	**Effect size**	
	**Mean**	**SD**	**Mean**	**SD**			**Cohen's *d***	**95% CI**
1. Mental illness is more common than…	0.63	0.49	0.78	0.42	1.86	0.07	0.27	0.16 to 0.66
2. The most common mental illness is…	0.22	0.42	0.61	0.49	4.32	<0.001	0.63	0.27 to 1.11
5. Mental illness most often begins when a person is…	0.72	0.47	0.84	0.4	1.30	0.20	0.21	−0.21 to 0.61
6. About one of _______ people develop a mental illness…	0.44	0.49	0.87	0.34	5.93	<0.001	0.80	0.26 to 1.11
10. What percentage of people with mental illness gets help?	0.26	0.43	0.54	0.5	3.49	<0.001	0.48	0.10 to 0.93
16. The mental illness of a person is likely caused by…	0.38	0.48	0.41	0.5	0.83	0.41	0.06	−0.35 to 0.47
20. In the U.S.A., more people go to the hospital because…	0.4	0.5	0.44	0.5	0.00	1.00	0.06	−0.35 to 0.47
21. People with mental illness often do fairly well in life…	0.46	0.51	0.85	0.36	4.12	<0.001	0.64	0.14 to 0.97
22. Parents can develop a mental illness when their kids…	0.34	0.47	0.52	0.51	2.14	0.04	0.29	−0.11 to 0.71
23. If someone in your family has a mental illness…	0.62	0.49	0.83	0.38	2.66	0.01	0.38	−0.07 to 0.76
30. Most people with mental illness are likely to get…	0.42	0.5	0.63	0.49	1.94	0.06	0.35	0.07 to 0.76
36. Epilepsy, or having seizures, is a mental illness…	0.5	0.51	0.67	0.48	1.64	0.11	0.27	−0.15 to 0.67
**Mental Illness subscale**	**5.35**	**1.9**	**7.96**	**2.04**	**7.4**	**<0.001**	**1.09**	**0.69 to 1.58**
3. One of the first steps to recovering from serious mental…	0.5	0.51	0.67	0.47	2.07	0.04	0.30	−0.12 to 0.70
7. Recovery from mental illness means…	0.35	0.48	0.39	0.49	0.47	0.64	0.06	−0.34 to 0.47
9. A lot of things can help a person recover from mental illness…	0.57	0.5	0.7	0.47	1.35	0.18	0.20	−0.22 to 0.60
13. One of the best combinations of things that can help people…	0.35	0.48	0.63	0.49	2.91	<0.01	0.43	0.02 to 0.85
15. Most people with mental illness…	0.44	0.5	0.44	0.5	0.00	1.00	0.00	−0.41 to 0.41
17. Family members can help a person with mental illness by…	0.28	0.46	0.59	0.5	3.18	<0.01	0.47	0.07 to 0.90
19. Getting help for mental illness works as well as getting help…	0.46	0.5	0.57	0.5	1.09	0.28	0.16	−0.24 to 0.57
28. If a person is trying to get attention by saying he or she will…	0.85	0.36	0.94	0.25	1.43	0.66	0.22	−0.22 to 0.60
31. Mental illness keeps a person from being able to develop…	0.53	0.5	0.8	0.4	2.60	0.01	0.40	−0.05 to 0.77
32. It is very important to know the cause of a person's mental…	0.2	0.4	0.24	0.43	0.63	0.53	0.09	−0.32 to 0.50
33. People with mental illness are usually able to make…	0.52	0.51	0.8	0.4	3.28	<0.01	0.48	0.02 to 0.84
35. Most people with a serious mental illness get well quickly…	0.85	0.36	0.89	0.31	0.63	0.53	0.09	−0.33 to 0.49
**Recovery subscale**	**5.89**	**2.06**	**7.65**	**1.84**	**5.87**	**<0.001**	**0.87**	**0.40 to 1.25**
4. The main reason people do not get help for their mental…	0.4	0.49	0.65	0.48	3.52	<0.001	0.46	0.04 to 0.87
8. People with mental illness…	0.52	0.51	0.74	0.44	3.16	<0.01	0.47	0.03 to 0.85
11. Some people think that people with mental illness are…	0.53	0.5	0.91	0.29	4.38	<0.001	0.67	0.13 to 0.96
12. Stigma toward people with mental illness happens when…	0.5	0.51	0.72	0.46	2.22	0.03	0.33	−0.10 to 0.72
14. People with mental illness…	0.35	0.48	0.59	0.5	2.69	0.01	0.40	−0.01 to 0.82
18. Most people with mental illness…	0.44	0.50	0.70	0.47	3.60	<0.001	0.53	0.10 to 0.93
24. Mental illness may be caused by being an emotionally…	0.33	0.47	0.61	0.49	2.93	<0.01	0.43	0.03 to 0.85
25. People with a mental illness are usually more dangerous…	0.67	0.47	0.89	0.32	2.66	0.01	0.40	−0.07 to 0.75
26. It is easy to tell if someone has a mental illness…	0.67	0.47	0.87	0.34	2.45	0.02	0.37	−0.09 to 0.74
27. People with a mental illness usually can't think well…	0.54	0.5	0.87	0.43	2.49	0.02	0.56	0.11 to 0.94
29. People with mental illness are more likely to be the victim…	0.48	0.51	0.37	0.49	−1.09	0.28	−0.16	−0.57 to 0.25
34. A person recovering from a mental illness is usually better…	0.7	0.47	0.87	0.34	2.43	0.02	0.35	−0.10 to 0.72
**Stigma subscale**	**6.12**	**2.23**	**8.67**	**2.1**	**7.14**	**<0.001**	**1.05**	**0.58 to 1.45**
Total KMIR scale	17.35	4.65	24.28	4.8	9.94	<0.001	1.47	1.03 to 1.95

*Full items can be made available by the first author. For completeness, changes in score by item have been included, however interpretation is recommended at the subscale and total scale level. Cohen's d effect sizes calculated after correcting for the correlation between pre and post-program scores, as per (84)*.

Youth reported how often they were able to use positive coping strategies across the program often (60.9%), sometimes (32.6%), and not very often (6.5%). Ninety three and a half percent of the youth said they were able to use positive coping strategies often or sometimes. Participant youth also rated their coping skills at the end of the program as compared to their skills at the beginning of the program; they said their coping skills were way better (51.1%), a little better (40.4%), and about the same (8.5%). Therefore, 91.5% of youth estimated their coping skills were way better or a little better from pre to post intervention.

The fidelity assessment revealed that fidelity objectives were met 94% of the time within the YES program delivery. For the 6% not met, it appeared that reduced session time available was a main barrier. Especially in the second semester and near holiday breaks, the schools sometimes shortened class hours to accommodate special activities such as pep rallies, standardized testing, and award ceremonies.

## Discussion

Data responded to the guiding research question: “What are the pre-post mental health literacy and coping outcomes reported by youth attending the Youth Education and Support program?” It appears that this sample of YES participant youth reported significantly increased levels of mental health literacy from pre to post program participation. Over 90% of the sample youth described the use of positive coping during the time they participated in the Youth Education and Support program. Over 90% of youth also reported improved coping skills from pre to post.

The YES mental health literacy outcomes align with the work of Kutcher et al. (64) who found participating in a mental health literacy program led to increased knowledge of mental health among Canadian high school students. The YES program youth participants showed less stigmatized assumptions from pre to post intervention. This result is similar to the work of Wahl et al. (66) in their implementation of a middle school mental health education curriculum. Since most mental health literacy programs are delivered to adults, this study may contribute to building knowledge about *youth outcomes* of mental health literacy programs. This is an important knowledge gap to address among mental health literacy program evaluations.

The Knowledge of Mental Illness and Recovery scale measuring levels of youth mental health literacy demonstrated promising psychometric properties including, reasonable model fit. Rasch analysis offers an approach to instrument development and validation that differs both conceptually and mathematically to the more commonly used classical test theory, where the aim is to measure across the range of the latent construct. Overall, the three subscales and the total scale score demonstrated adequate or good fit to the model. Reliability coefficients were generally fair for the subscales, and considered good for the total scale.

Item redundancy were not found to be an issue for any item pair across all KMIR subscales. Given the relatively small number of items in each scale, this is somewhat expected, as the probability of redundant items increases with the number of items given that there are a finite number of ways in which a single construct can be represented in written language. However, even small numbers of items are not immune from the effects of redundancy, with items that are too similarly worded or of very similar difficulty likely to result in correlated residuals after accounting for the latent constructs. As noted earlier, differential item functioning was not explored given the sample size. Local independence was observed for several item pairs; however, given that there were no significant gains to be had from the removal of dependent items, all were retained.

There remain a number of areas for improvement, and it is important to note that the KMIR scale is still under development. Data collection is underway to measure test-retest and test convergence analysis of the KMIR compared to the Wahl et al. (66) knowledge scale. The development of scales with adequate psychometric properties is a key need for the development of evidence-based mental health literacy programs for youth.

The YES program does provide an early response to the requests for more mental health information echoed among youth that have a parent or other family member with a mental health disorder (57). The active learning format of the YES program and the readability of the KMIR scale seems aligns with the recommendation of parent mental health consumers for delivering mental health literacy programs that are fun and developmentally appropriate (85).

The YES evaluation includes youth-estimated coping skills is useful in beginning to collect behavioral change data. This is important since resiliency and coping theories include a need for knowledge and behavioral foci. The YES small group format may have provided an additional support network for the youth. Support networks are key components within resiliency and coping theories (11, 49).

However, it is not possible, even for this sample of youth, to know the extent that participation in a mental health literacy program may serve as a developmental factor in the prevention of mental health disorders or their reduction in symptoms severity. The YES program participant sample is relatively small and the KMIR scale is still undergoing psychometric testing. Family mental health disorders reported by parents were not subject to professional confirmation. Most critically, the study did not use a control group. It is likely that having the YES qualitative data analyzed by outside reviewers may have enhanced findings validity; an audit of the data using the trails of a codebook and raw data would also have contributed an external review of analyses (86). The findings cannot be generalized to all children of a parent or other family member with a mental illness.

There is a need for more funding, including long-term evaluation of mental health programs for youth. Specialized curricula may be needed for children that have a parent or other family member with a mental health disorder (4). The voices of youth, parents, and other family members should be included in the program development and evaluation. Intergenerational dialogue among family members may be helpful (87). Future research should include studies with matched comparison or control groups. The use of a randomized wait list controls may be an option. The inclusion of additional standardized scales to examine youth, parent, and family functioning would provide a stronger picture for assessing pre, post, and follow up intervention.

Mental health disorders one of the most common sources of disability in the world and mental health literacy may be able to build human developmental resiliency. Therefore, federal and state governments, as well as private funders of mental health research, should fund mental health literacy programs. Seeking this funding will require policy advocacy on the part of community members (30). It makes sense to build and test mental health literacy programs for youth, parents, and intergenerational family members (24, 88) to explore what works for whom, when, how, and for how long.

Despite many limitations, this study offers hope in findings that youth participating in a mental health literacy program reported increased levels of mental health literacy and coping skills. The target group was largely composed of youth that reportedly had a parent with a mental health disorder. The work is likely a small step of progress toward reducing the knowledge gaps for building evidenced-based mental health literacy programs for youth at risk of acquiring a mental health disorder. To do so, offers even more hope for a future world where mental illness is discussed respectfully, professional help is forthcoming, and accurate information about mental health is widely available.

## Ethics Statement

The study was carried out in accordance with the recommendations of the human subject guidelines of the Michigan State University Institutional Review Board. The protocol was approved by the committee. All subjects gave assent (if minors) and their parents gave written consent.

## Author Contributions

JR lead the project and worked on all sections of the paper. SC author led the norming of the scale and wrote Tables 1–3. DC wrote the background section on scales and the information on resiliency and also coded all the data from which the paper was developed. CG wrote the background on children at risk and added to the discussion section. All authors helped with reviewing drafts.

### Conflict of Interest Statement

The authors declare that the research was conducted in the absence of any commercial or financial relationships that could be construed as a potential conflict of interest.
